# The Effect of Cyber-Ostracism on Social Anxiety Among Undergraduates: The Mediating Effects of Rejection Sensitivity and Rumination

**DOI:** 10.3390/bs15010037

**Published:** 2025-01-01

**Authors:** Chun Shi, Peizhen Sun, Jiaru Shi, Haosheng Ye, Junyan Tao

**Affiliations:** 1 School of Education, Guangzhou University, Guangzhou 510006, China; 2 Department of Psychology, School of Education Science, Jiangsu Normal University, Xuzhou 221116, China

**Keywords:** cyber-ostracism, social anxiety, rejection sensitivity, rumination

## Abstract

In order to examine the relationship between cyber-ostracism and social anxiety among undergraduates, as well as to investigate the mechanisms underlying the effects of cyber-ostracism, rejection sensitivity, and rumination on social anxiety, this study recruited 864 undergraduate students from Jiangsu and Guangdong Provinces in China using a cluster sampling method. The participants completed the Cyber-Ostracism Questionnaire, the Chinese version of the Interaction Anxiousness Scale, the Chinese version of the Rejection Sensitivity Questionnaire, and the Chinese version of the Ruminative Responses Scale. Furthermore, the mediating effects were examined using the structural equation modeling (SEM) method. The results showed that (1) cyber-ostracism was positively related to social anxiety among undergraduates, and (2) rejection sensitivity and rumination played a chain mediating role in the relationship between cyber-ostracism and social anxiety.

## 1. Introduction

Compared with high school students, who rely more on reality and intuitive experience in their learning and communication, undergraduate students tend to adopt more virtual platforms and digital technology in their lives. Therefore, internet technology has a significant impact on the learning, communication methods, and lifestyles of undergraduate students. Individuals can maintain their existing relationships while establishing new ones without being constrained by distance. The convenience and real-time interactivity of this kind of online communication can, to a certain extent, satisfy the social needs of college students. Conversely, the high degree of involvement in online social interactions can also increase the chance of individuals being ignored and excluded in the network. This phenomenon is called cyber-ostracism (Williams et al., 2002), which refers to the extension and development of real social exclusion in network social situations (Schneider et al., 2017). At present, society is in an Internet era and, with the development of Internet technology, cyber-ostracism is no longer limited to a specific online community or platform, but widely exists in various social media platforms, online forums, online game communities, and other online spaces. Cyber-ostracism may trigger a series of chain reactions, resulting in harm towards the harmony and stability of both online and real society.

Cyber-ostracism results in a series of negative influences on an individual’s psychological health. Former studies have found that, compared to ostracism in the real world, cyber-ostracism has a wider scope, a stronger concealment, and a more severe impact on the psycho-social adaptation of individuals (Sun et al., 2017). There are many carriers of cyber-ostracism, such as online group chats, social media platforms, e-mail, video games, and so on (Williams, 2007; Tobin et al., 2015). Negative emotional experiences of cyber-ostracism can be caused by situations such as not receiving replies in group chats or lack of comments in one’s posts on social media platforms; in this way, ostracism has become more common in the online environment. In the Chinese context, undergraduate students are the main Internet users, and their study, life, and way of thinking are inevitably affected by the Internet. Furthermore, new undergraduate students represent a decisive period of development, bridging the gap between the latter part of their adolescence and the onset of adulthood (You et al., 2016). During this period, their psychological condition can be particularly sensitive. Studies have pointed out that undergraduate students will exhibit stronger anger and hostility when they are facing exclusion, thereby triggering aggressive behaviors (J. Liu et al., 2018). Various studies have been conducted in recent years to explore the effects of cyber-ostracism on the psychological health and behavioral patterns of undergraduate students (Jin et al., 2019; Shi et al., 2023; Lei et al., 2018). Empathizing with the impact of cyber-ostracism on undergraduate students is of great significance for maintaining their development of mental health.

### 1.1. Cyber-Ostracism and Social Anxiety

Cyber-ostracism is closely related to negative emotions. Robert Agnew—the proponent of general strain theory—pointed out that the negative relationships between an individual and others are the essential cause of stress and pressure (Agnew, 1992). When an individual has excessively high expectations of others but does not receive the corresponding treatment, it can provoke a range of adverse emotional responses, including despondency, depression, anxiety, aggressiveness, and so on (Yang, 2003). Relevant research has found that individuals who experience cyber-ostracism exhibit negative emotions (Williams et al., 2002), with social anxiety being a common negative emotional experience. Studies have shown that, when individuals experience cyber-ostracism, they tend to experience more anxiety (Heeren et al., 2012). Baumeister and Tice also discovered that anxiety is the primary response of individuals to social exclusion (Baumeister & Tice, 1990). Drawing on the literature above, we hypothesized the following:

**H1:**  *There is a positive correlation between cyber-ostracism and social anxiety.*

### 1.2. The Mediating Role of Rejection Sensitivity and Rumination

In Williams’ temporal need–threat model (Williams, 2009), individuals who experience social exclusion undergo three distinct phases, each marked by varied psychological responses. During the reflexive stage, the excluded individual reacts instinctively—a response that is largely independent of individual differences and situational factors. The second phase is the reflective stage, which involves an assessment of the cause and significance of social exclusion. In this stage, both situational factors and individual differences influence cognitive evaluation, which subsequently shapes behavioral responses in the withdrawal stage. According to this model, an individual’s reaction to cyber-ostracism is influenced, on one hand, by the characteristics of the online context (e.g., its uncertainty, ambiguity, disembodiment, and variability) and, on the other hand, by individual differences, including personality traits and cognitive styles.

Rejection sensitivity is a personality trait that denotes an individual’s excessive worry and negative anticipation about potential rejection, exclusion, or non-acceptance in social interactions (Mehrabian, 1970). Elevated rejection sensitivity plays a key role in the genesis of anxiety disorders (Nelson & Hajcak, 2017) Individuals characterized by heightened rejection sensitivity are more likely to perceive ambiguous or unintelligible information from others in social exchanges as indications of rejection, resulting in negative emotions such as anxiety (Downey et al., 1998). Buckley et al. discovered that rejection sensitivity is strongly associated with an individual’s reactions to interpersonal rejection and acceptance, but it does not play a moderating role in these reactions (Buckley et al., 2004). From the perspective of evolutionary psychology, Kerr and Levine have argued that humans’ sensitivity to the threat of interpersonal rejection has deep evolutionary significance (Twenge et al., 2007). The social monitoring system theory suggests that individuals possess a keen ability to detect social rejection in online contexts (Cheng et al., 2014). In online social interactions, individuals with high rejection sensitivity are prone to anticipating rejection, even in neutral or uncertain online social situations. They tend to excessively worry about being rejected by others, resulting in adverse emotional responses including depression and anxiety. Drawing on the literature above, we hypothesized the following:

**H2:**  *Rejection sensitivity plays a mediating role between cyber-ostracism and social anxiety*.

Rumination is a learned mode of thinking categorized as a cognitive bias (Cheng et al., 2014), where an individual unconsciously and persistently focuses on their emotions, behaviors, and thoughts, engaging in continuous and repetitive contemplation of their possible causes, resulting consequences, and event details, rather than actively seeking solutions. As a cognitive style that repeatedly attends to one’s negative emotions and related events, we developed the following hypothesis:

**H3:**  *Rumination plays a mediating role between cyber-ostracism and social anxiety.*

Previous studies have demonstrated that individuals exhibiting high rejection sensitivity are more prone to engage in ruminative thinking (Papageorgiou & Siegle, 2003). Those with heightened rejection sensitivity are more easily trapped in ruminative thoughts which, in turn, lead them to pay greater attention to negative events, thereby exacerbating their anxiety. Drawing on the literature above, we hypothesized the following:

**H4:**  *Rejection sensitivity and rumination play a chain mediating role between cyber-ostracism and social anxiety.*

### 1.3. Current Study

In recent years, the adverse impacts of cyber-ostracism on the social anxiety of individuals have attracted attention from academia. Based on the temporal need–threat model and relevant empirical research findings, an individual’s response to cyber-ostracism can be affected by online environments and personal traits. However, while former studies have placed more attention on the influential results of cyber-ostracism on social anxiety, there is limited research on the mechanisms driving the relationship between these two factors; moreover, rejection sensitivity and rumination may play bridging roles in the relationship between ostracism and social anxiety. This study aimed to explore the mechanism underlying the impact of cyber-ostracism on social anxiety, revealing the important roles of rejection sensitivity and rumination in this process, which not only enriches the field of cyber-ostracism and social anxiety, but also provides theoretical support for the advancement of psychological well-being interventions. This research also paves the way for innovative methods within the realm of future psychological studies. In addition, this study adopts a large sample questionnaire-based approach to ensure the reliability of the data, followed by an in-depth exploration of the mediating roles of rejection sensitivity and rumination between cyber-ostracism and social anxiety, providing a new perspective on the problem of psychological well-being in online environments.

## 2. Materials and Methods

### 2.1. Participants and Procedure

A cluster random sampling method was utilized to select university freshmen from both Xuzhou, Jiangsu Province, and Guangzhou, Guangdong Province, as the research participants. All participants were gathered in classrooms to complete the questionnaire, as this approach for collecting data can ensure the homogeneity of the environment and the standardization of the process, as well as ensure that participants fill out the questionnaire carefully, among other advantages. The participants were clearly informed that their answers would only be used for research purposes and were promised that the principles of voluntary participation, anonymous processing, and confidentiality would be followed. Before the participants officially started to complete the questionnaire, the experimenters provided detailed instructions regarding the questionnaire; for example, that the questionnaire would take around 20 min to complete and that all participants were required to complete the questionnaire independently. All participants signed informed consent forms, confirming that they fully understand the purpose and process of the study. The methods carried out throughout this research followed the principles written in the 1964 Helsinki Declaration, including subsequent revisions, and complied with the ethical guidelines set by the Institutional Review Board of Jiangsu Normal University.

The survey was conducted collectively with classes as the unit, and 1042 questionnaires were distributed. Questionnaires with an answer time of less than 180 s and those with completely identical responses were excluded. Ultimately, after the data collection process, a total of 864 valid questionnaires were retained, yielding a response rate of 82.9%. The average age of the participants was 18.8 years old (SD = 0.8 years). Among them, there were 269 males and 595 females, with 319 students majoring in science and engineering, 323 students majoring in liberal arts, and the remaining 222 students majoring in arts and sports.

### 2.2. Measures

#### 2.2.1. Cyber-Ostracism

The Cyber-Ostracism Questionnaire developed by Tong (2015) was adopted, consisting of 14 items rated on a 5-point Likert scale. It encompasses three dimensions: cyber-ostracism in online interpersonal chat, in online group chat, and in personal cyberspace. An elevated total score signifies greater exposure to cyber-ostracism on the part of the participants. The Cronbach’s α of the questionnaire in this study was 0.96.

#### 2.2.2. Social Anxiety

The Interaction Anxiety Scale (IAS) developed by Leary and modified by Wang et al. (1999) was adopted (Peng et al., 2004), consisting of 15 items rated on a 5-point Likert scale. Items 3, 6, 10, and 15 are reverse-scored. An elevated total score indicates a greater severity of social anxiety. The Cronbach’s α of the scale in this study was 0.86.

#### 2.2.3. Rejection Sensitivity

The Rejection Sensitivity Questionnaire (RSQ) developed by Downey and Feldman and revised by Zhao et al. (2012) was adopted, consisting of all 18 items rated on a 6-point Likert scale. This scale encompasses a range of daily routes through which undergraduate students might seek assistance, prompting participants to conceive asking for help and to predict the reactions they might receive. Each item contains two questions: the first assesses the anxiety level towards rejection from significant others, with a higher score indicating greater anxiety; the second assesses the expectation of acceptance from significant others, with a higher score corresponding to a higher anticipation of acceptance. The rejection sensitivity score is calculated as total score = anxiety degree score × acceptance expectation degree reverse score, with an elevated total score corresponding to higher rejection sensitivity. The Cronbach’s α of the scale in this study was 0.92.

#### 2.2.4. Ruminative

The Ruminative Responses Scale (RRS) developed by Nolen-Hoeksema and revised by Han and Yang (2009) was adopted, consisting of 22 items rated on a 4-point Likert scale. It comprises three dimensions: symptom rumination, reflective pondering, and brooding. A higher score indicates a higher level of rumination. The Cronbach’s α of the scale in this study was 0.94.

### 2.3. Data Analysis

Descriptive statistics, common method bias test, and correlation analysis were conducted utilizing the SPSS 26.0 software, while we used Amos 16.0 to perform the mediation model test.

## 3. Results

### 3.1. Common Method Biases

Harman’s one-factor test was adopted to assess the common method bias. The results indicated that 14 eigenvalues greater than 1 were obtained without rotation, where the first factor explained only 22.17% of the total variance, falling below the critical threshold of 40%. These results suggest no substantial common method biases in this study.

### 3.2. Descriptive Statistics and Correlations for Variables

A correlation analysis was conducted on cyber-ostracism, social anxiety, rejection sensitivity, and rumination. The results of the correlational analysis showed that cyber-ostracism exhibited significant positive correlations with rejection sensitivity, rumination, and social anxiety. Rejection sensitivity demonstrated a significant positive correlation with rumination and social anxiety, while rumination was positively correlated with social anxiety. The mean, standard deviation, and correlation matrix for each variable are provided in Table 1.

### 3.3. The Mediating Role of Rumination and Rejection Sensitivity Between Cyber-Ostracism and Social Anxiety

An investigation of the total effect of cyber-ostracism on social anxiety was conducted, using a model constructed on the basis of cyber-ostracism as the independent variable and social anxiety as the dependent variable (Model 1). The results indicated a high degree of correspondence between the model and the data (x^2^ = 4.35, x^2^/df = 4.35, CFI = 0.99, TLI = 0.99, SRMR = 0.01, RMSEA = 0.06). The regression coefficient of cyber-ostracism on social anxiety was significant (β = 0.22, *p* < 0.001), thereby indicating that cyber-ostracism exerted a significant total effect on social anxiety.

**Figure 1:**
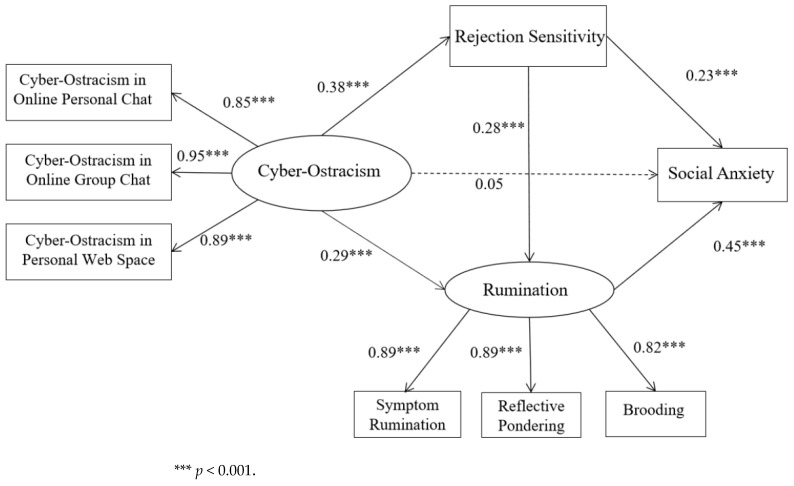
Model of the chain mediation effect between cyber-ostracism and social anxiety.

Furthermore, this study explored the mediating roles of rejection sensitivity and rumination in the relationship between cyber-ostracism and social anxiety. The study’s analysis positioned cyber-ostracism as the independent variable, society anxiety as the dependent variable, and rejection sensitivity and rumination as the mediating variables, in accordance with Model 2. The Amos 26.0 software was utilized to test the mediating effects. The results indicated that the fit of this model was acceptable (χ^2^ = 116.13, χ^2^/df = 7.26, RMSEA = 0.09, CFI = 0.98, GFI = 0.97, AGFI = 0.93, IFI = 0.98, NFI = 0.97, TLI = 0.96, SRMR = 0.03). Figure 1 shows the results of the mediating effect model. The bias-corrected bootstrap method was adopted, with 5000 repeated samples, in order to calculate the 95% confidence interval of the mediating effect; in particular, when 0 was not included in the interval, it indicated that the indirect effect was significant. The results indicated that cyber-ostracism significantly influenced social anxiety via the pathway of sensitivity (95% CI = [0.08, 0.10]), and that the impact of cyber-ostracism was also significantly mediated by rumination (95% CI = [0.11, 0.15]). Furthermore, the serial mediation effect was also significant (95% CI = [0.04, 0.06]). The path effect values and 95% confidence intervals of the chain mediation model are shown in Table 2.

## 4. Discussion

### 4.1. Theoretical Implications

Consistent with our hypothesis 1, the results revealed that cyber-ostracism is significantly and positively correlated with social anxiety, aligning with previous findings (Z. Q. Liu, 2022) and supporting the broader perspective of general strain theory (Sun et al., 2017). As the frequency of online social interactions among university students continues to escalate, the uncertainty, ambiguity, and unconstrained nature of these interactions can easily lead individuals to perceive that they are not being treated as expected in these relationships, fostering experiences of cyber-ostracism, which is subsequently associated with increased anxiety. Consequently, cyber-ostracism emerges as a crucial risk factor associated with social anxiety among university students. Furthermore, our study indicated that the connection between cyber-ostracism and social anxiety can be mediated through three different approaches.

Consistent with the proposed hypothesis 2, this study revealed that rejection sensitivity serves as a mediator in the association between cyber-ostracism and social anxiety, consistent with previous research (Downey et al., 1998; Butler et al., 2007). The Rejection Sensitivity Model (Ayduk et al., 2001) posits that those people who have experienced rejection enter a state of heightened vigilance, which is tied to anxious or hostile expectations of further rejection. These expectations may distort an individual’s perceptions of the words and actions of others, increasing their defensiveness. This anxious anticipation can lead to a self-fulfilling prophecy, where an individual’s fears of issues arising in online social situations materialize, perpetuating a cycle of rejection. Consequently, individuals with high rejection sensitivity are highly sensitive to cues of rejection in their environment. Coupled with the ambiguity of online social settings, such as the absence of physical presence, the constant shift between online and offline status, incomplete or relatively unconstrained language expressions, and other vague information, these cues are often interpreted by highly-rejection-sensitive individuals as rejection, thereby eliciting anxiety.

Consistent with the proposed hypothesis 3, this study also revealed that rumination serves as a mediator in the association between cyber-ostracism and social anxiety, consistent with recent related research perspectives (Huang, 2020). The Response Styles Theory indicates that rumination is a maladaptive response style that plays a significant role in initiating, maintaining, and accelerating social anxiety (Valenas & Szentagotai-Tatar, 2015; Fang & Sun, 2018), significantly predicting social anxiety. After experiencing cyber-ostracism, individuals develop conflicting beliefs about online social interactions, leading to immense psychological pressure and triggering rumination. As they repeatedly contemplate the events themselves and their outcomes, their cognitive resources become unable to shift away from negative events, hindering constructive actions. This exacerbates negative self-perceptions, emotional regulation difficulties, and other issues, ultimately inducing social anxiety.

This study demonstrated that rejection sensitivity and rumination play a sequential mediating effect in the relationship between cyber-ostracism and social anxiety, which is consistent with our hypothesis 4. Previous research has indicated that people with elevated rejection sensitivity exhibit a heightened awareness of signals of being rejected within online interpersonal settings, even interpreting ambiguous or uncertain disclosures as rejection (Luo, 2017; Guo, 2021). The temporal need–threat model (Williams, 2009) posits that, during the reflection phase, situational factors and individual differences influence cognitive appraisal. The uncertainty and real-time dynamics of online communication contexts (situational factors), high rejection sensitivity (personality trait), and high rumination (thought pattern) are crucial situational and individual factors that affect an individual’s cognition of events and online social environments, their judgments about future event developments, and the induction of social anxiety. High rejection sensitivity not only influences an individual’s judgments about the future development of events, but also impacts the events themselves. In other words, it may exacerbate their negative cognitions, making them more likely to engage in negative re-experiencing of events, fall into maladaptive rumination, and decrease their emotional regulation abilities, thereby increasing the likelihood of social anxiety.

This study investigates the current status and influencing mechanisms of social anxiety among college students, examining the predictive effects of cyber-ostracism on social anxiety with rejection sensitivity and rumination as the mediators. Theoretically, it integrates and validates the assumptions of general strain theory and the temporal need–threat model, enhancing our understanding of the roles of rejection sensitivity and rumination. It confirms the hypothesis of multiple mediating mechanisms in the relationship between cyber-ostracism and social anxiety, further refining the theoretical model.

### 4.2. Implications for Practice

In practice, the results of this study revealed the formation pathway of social anxiety, highlighting how cyber-ostracism significantly influences social anxiety. Considering that a high frequency of online social interactions increases the chances of ignorance and exclusion of individuals online (Williams et al., 2002), we believe that it is essential to foster an inclusive and respectful online interaction environment that can encourage people to treat others with kindness and rationality while reducing the occurrence of offensive or insulting remarks, thereby lowering the risk of cyber-ostracism. In addition, this study delved into how two mediators—rejection sensitivity and rumination—mediate the connection between cyber-ostracism and social anxiety, providing empirical support for guidance regarding the interpersonal communication of undergraduate students. For undergraduate students who suffer from cyber-ostracism, on one hand, we can help them to use self-regulation and self-affirmation strategies (Zhang & Xiao, 2018), adopting psychological intervention methods such as self-awareness, reflection, supervision, and regulation of their social interaction activities to conduct self-adjustment (Lin et al., 2023) in order to alleviate rejection sensitivity, thereby reducing social anxiety. On the other hand, it is advisable for undergraduate students to use online social networking rationally, avoid investing excessive cognitive resources into online social interactions, reduce the generation of ruminative thinking (Cao et al., 2023), and reduce social anxiety. Through implementing the abovementioned measures, we can help undergraduate students to establish a positive cognitive pattern for online interaction, thereby interrupting the induction and reinforcement process of social anxiety while promoting healthy interpersonal communications.

### 4.3. Limitations and Future Research Directions

This research, however, is not without its limitations, which are as follows: First, this study adopted a cross-sectional design that allowed us to collect a large amount of data at one time point and assess the correlations between variables at that time point; however, it is challenging to determine the causality between variables when adopting a cross-sectional approach. Further studies can consider adopting longitudinal approaches to conduct long-term tracking of the same group of individuals and explore the causal relationships between the variables. Second, the scope of the sample selection was also limited, as only students from two universities were selected as participants. Such a limitation may result in constraints in the universality and extensibility of the research findings. Research in the future can be further expanded to include other student groups (e.g., high school students, graduate students), as well as non-student groups (e.g., working professionals, retirees, and people of different socio-economic statuses). Such expansions will contribute to a more comprehensive understanding of the impact and mechanisms of cyber-exclusion on social anxiety. Third, this study did not explore precise measurement of the specific occurrence time of cyber-ostracism. Due to the dynamic nature of cyber-ostracism, it may be influenced by multiple factors and exhibit different characteristics at different time points. Therefore, our study may not have been able to capture all the details and changes of cyber-ostracism. Future studies can adopt more refined time measurement approaches, such as real-time data collection or longitudinal study design, in order to gain a more accurate understanding of the process of cyber-ostracism. Moreover, in future studies, we will continue to explore the moderating effects of gender, culture, and previous experiences of social exclusion on cyber-ostracism, with the aim of gaining a more comprehensive understanding of this complicated social phenomenon. Our research aims to furnish a scientific basis for the creation of influential psychological intervention approaches, promoting the healthy development of cyberspace while ensuring the mental health of individuals.

## Figures and Tables

**Table 1:** Correlation between variables.

Variables	M	SD	1	2	3	4
1. Cyber-Ostracism	1.87	0.68	1			
2. Social Anxiety	3.23	0.63	0.20 **	1		
3. Rejection Sensitivity	9.83	4.74	0.37 **	0.38 **	1	
4. Rumination	2.17	0.53	0.39 **	0.49 **	0.38 **	1

Note. ** *p* < 0.01.

**Table 2:** Results of bootstrap analysis for significance test of mediation effect.

Path	Effect Size	95% CI	
		Lower	Upper
Cyber-Ostracism → Rejection Sensitivity → Social Anxiety	0.09	0.08	0.10
Cyber-Ostracism → Rumination → Social Anxiety	0.13	0.11	0.15
Cyber-Ostracism → Rejection Sensitivity → Rumination → Social Anxiety	0.05	0.04	0.06

## Data Availability

The raw data supporting the conclusions of this article will be made available by the authors, without undue reservation.
